# A pre-failure narrow concrete cracks dataset for engineering structures damage classification and segmentation

**DOI:** 10.1038/s41597-023-02839-z

**Published:** 2023-12-21

**Authors:** Karolina Tomaszkiewicz, Tomasz Owerko

**Affiliations:** AGH University of Krakow, al. A. Mickiewicza 30, 30-059 Krakow, Poland

**Keywords:** Civil engineering, Research data, Environmental impact

## Abstract

Monitoring of structures’ condition plays a fundamental role in providing safety for users and extending the structures’ lifespan. The monitoring is conducted through on-site inspections by engineers thus this process is time-consuming, labor-intensive and prone to subjective engineering opinions. Detecting damage using machine learning algorithms on images can support engineers’ work, especially for early damages which are difficult to see with the human eye. This article is focused on the concrete crack detection problem in engineering structural elements. Despite the availability of several concrete crack detection datasets, no dataset allows semantic segmentation of cracks narrower than 0.3 mm (the crack width limit for typical engineering structures elements and environmental conditions according to EC 1992-1-1) and the ability for crack classification is limited. The provided open dataset represents only cracks below the crack width limit of 0.3mm, which do not yet indicate concrete elements failure. It is dedicated for early crack classification and segmentation, so that damage protection can be taken at an early stage to prevent structural element damages.

## Background & Summary

Assessing and monitoring the concrete structure condition play a fundamental role in providing safety for its users, maintaining the utility functions of the object and extending the life of the structure. This makes it possible to realize activities for sustainable development, such as reducing the carbon footprint of road and rail infrastructure, and re-using structural elements as a result of keeping them in good condition. This paper focuses on the problem of crack detection for concrete elements of engineering structures. It is one of the more difficult aspects in the diagnosis of concrete structures due to the very low limits of acceptable damage and the far-reaching consequences for the damaged structure.

Observation of the appearance and possible propagation of concrete element cracks has a significant role in the process of monitoring the technical condition of concrete structures. Concrete cracks are caused when the tensile stress at a given point in the structure exceeds the tensile strength of the concrete. The appearance of cracking is a natural phenomenon occurring in concrete structures. It is necessary to ensure that the width of cracks that occur at the stage of construction and use of the structure does not exceed the acceptable values in accordance with the design of the structure and the applicable standard values for the country (such as those included in EC 1992-1-1^1^ or national standards). At the same time, it is important to determine the most probable cause of the cracks. This allows to them to decide correctly on the necessary scope and urgency of the repair and protection actions. For this reason, not every crack will indicate a risk to the structure. The correct classification of damage should be done by an experienced engineer who knows how to correlate the working conditions of the structure and the environmental conditions with the time and location of damage appearance. In addition, crack detection is a difficult problem due to the very narrow limit of damage allowed and the far-reaching consequences for the damaged structure if this value is exceeded.

Inspections of construction structures are required by relevant national regulations. A different approach is used in the Authors’ country that is Poland^2–4^, and others in European countries^5,6^, or non-European countries^7–10^. These regulations, depending on the type of construction, determine the frequency of inspections and their scope. It should be pointed out that inspections of construction structures are still mainly realized by inspectors, who personally verify the appearance of damage to individual elements of the structure, assess their condition and decide on the scope and urgency of the necessary repairs. This process is labor-intensive, time-consuming and is dependent on the inspector’s subjective opinion depending on the engineer’s experience. It is estimated that about 50% of the given condition assessments are incorrect or vary from inspector to inspector^11^.

Every year, the number of bridges that are in deteriorating condition increases. It seems rational to invest more in monitoring the growing number of aging structures and detecting damage at an early stage. 38% of bridges (224,000 spans) in the U.S. need repair, 78,800 bridges should be replaced. More than 43,500 bridges are in poor condition and have been classified as “structurally deficient”^12^. The same report indicates that at the current rate, it would take nearly 30 years to repair these structures^12^.

Detecting and observing narrow cracks in time, i.e. at an early stage of their formation, makes it possible to protect the damage and slow down its propagation so as to prevent the structure’s progression to failure. Proper protection of the damage also reduces the possibility of other damage, such as the possibility of water penetrating deep into the structure in the location of the crack, and thus the risk of reinforcing bar corrosion and material degradation. Crack detection in a pre-failure state not only reduces repair costs, but most importantly reduces the number of bridges out of service. The importance of the problem can be illustrated by reports that, using the US as an example: 43,500 bridges classified as “structurally deficient” drivers cross 167.5 million times a day^12^. It should be noted that bridges are one of the critical infrastructures of countries and have an important role in the aspect of defense. All this underscores the need to detect damage at the earliest possible stage, both in terms of financial, social and security aspects.

Narrow cracks in this paper are understood as cracks with a width not exceeding the permissible limit of 0.3mm according to EC 1992-1-1^1^. EC 1992-1-1^1^ defines the limit widths of cracks depending on the type of element (i.e., reinforced members and prestressed members with unbonded tendons or prestressed members with bonded tendons) and the exposure class, i.e., the environmental conditions under which the structure is working.

EC 1992-1-1^1^ identifies three values, that is, 0.2mm, 0.3mm and 0.4mm. For the dataset presented here, the assumption was made to consider only those cracks whose width does not exceed 0.3mm. This value corresponds to elements reinforced and prestressed with unbonded tendons and typical environmental conditions (expressed as exposure classes) in which bridge structure elements are working.

By detecting the crack in the pre-failure stage, it is possible to start monitoring the progression of the damage, as well as to carry out the necessary protection or repair work. At the same time, this is the stage when cracks are potentially much more difficult to recognize with the human eye than cracks of wider width. For this reason, in many cases the inspector may not identify such damage at an early stage. As a support for the inspector’s work, deep learning algorithms can be used for the identification of structural cracks on images. It should be pointed out that algorithms dedicated to the problem of cracks detection based on classical machine learning methods (i.e. not deep learning), e.g. edge filters, do not provide good results for each width of the crack. Very often they make mistakes due to the presence of dirt, shading and differences in the texture of concrete on the surface recognizing them as cracks.

Currently available datasets are dedicated to solving the problems of computer vision and machine learning for cracks of wider width, which can indicate the failure status of structure. Many satisfactory results have been achieved both in the area of crack detection using image processing techniques and machine learning methods, as well as algorithms which enable the inspection of crack course, its length and width^13–15^.

Even though it is possible to have good measuring equipment to take images specific to the considered problem, the dataset creation for the pre-failure condition requires to fulfill several additional prerequisites. The actual measurement material acquired in the process of a formal bridge inspection procedure is needed^2–10^. The person creating the masks must have a high degree of content knowledge to identify the damage the way not to confuse the cracking resulting from the pre-failure condition with other sources of cracking of the concrete surface such as dirt, etc. The interpretation of a crack as potentially leading to failure of a bridge structure in the future must be related to the nature of the element’s work and the point in the structure’s life cycle at which the damage occurred.

Despite the increasing number of datasets dedicated to structural damage detection using machine learning^16^, at the moment there are no open datasets dedicated to solutions based on semantic segmentation relating to narrow cracks, and the number of classification datasets is strongly limited. By “open datasets” the Authors mean such datasets that can be downloaded without contacting the author. Thus, the available datasets do not solve the key problem from the bridge engineer’s point of view, which is creating solutions using machine learning for pre-failure states.

In the context of classification, mention should be made of the SDNET^17^ dataset, which admittedly includes cracks with widths ranging from 0.06mm to 25mm. It means that this dataset includes a set of damage that does not exceed the limit of 0.3mm, as well as cracks indicating that the failure states of the structure have been exceeded. However, the dataset’s authors do not extract and specify the set size in terms of individual damage widths, and also do not assign damage causes to the images. The KrakN dataset^18^, on the other hand, contains cracks with a width of up to 0.2mm, but is based only on images from a single bridge pillar, which is not representative of the wide range of possible damaged elements and causes of damage.

As the authors’ experience shows, the use of available datasets and the use of transfer learning to train a crack detection network does not provide correct results for narrow cracks^19^. This solution is successful only for such images when the size of the crack in the tested image is similar to the size of the crack in the images used to train the network^19,20^.

**Fig. 1:**
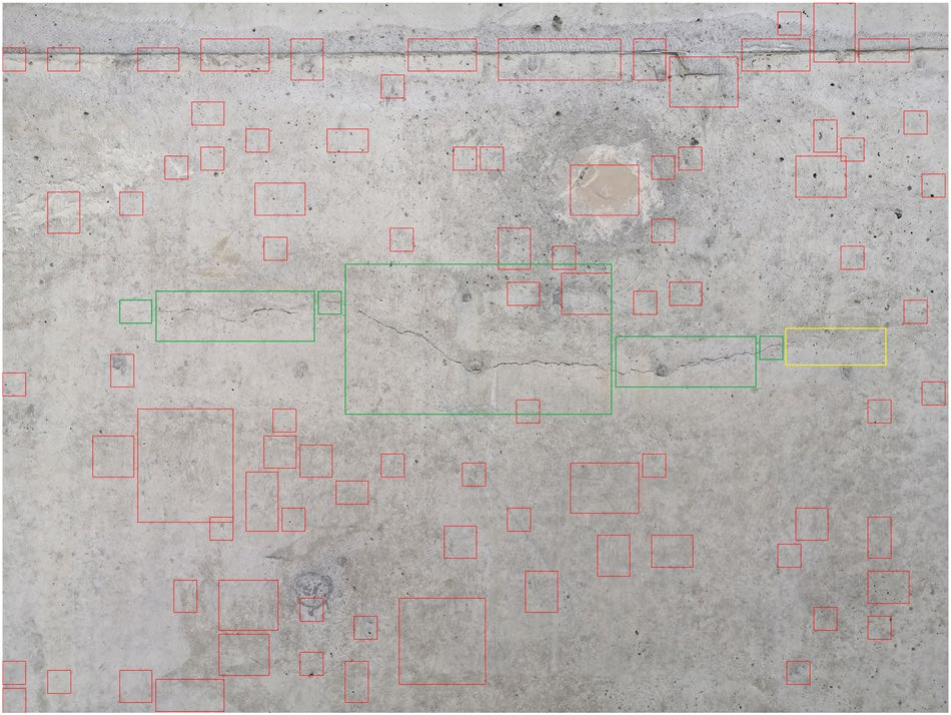
Limitations of using the network trained on the KrakN dataset for the case of narrow cracks. Green rectangle - cracks correctly detected, red rectangle - detection of a cracks by the network in a place where they do not actually occur, yellow rectangle - a crack not detected by the network, despite the fact that it occurs in that place.

The authors also conducted an experiment using weights of the network trained on narrow cracks^21^ using images from the KrakN^18^ dataset. The solution’s effectiveness was verified on one of the images, which was later used to build a dataset for the narrow cracks. It was shown that the solution’s based on automatic methods and an undifferentiated dataset (i.e. KrakN^18^ including images of only one pillar) does not support the bridge engineer’s work. The number of incorrect identifications - as shown in Fig. 1 - does not allow the use a network trained in this way to support the inspection process.

Based on this, it can be concluded that crack classification on images at the stage when their width does not exceed the permissible limit is strongly limited, and their segmentation is not possible (https://www.ck.gov.pl/review/id/54326/type/ud.html). As a consequence, it is not possible to diagnose and monitor the progression of cracks at an early stage of their formation using the open datasets available so far.

The dataset presented in this paper addresses a diagnosed gap in the field of publicly available datasets for problems related to the use of deep learning for semantic segmentation and classification of narrow cracks, characterizing the pre-failure condition of engineering structure concrete elements. This dataset is dedicated to all researchers working in the field of computer vision, machine learning and deep learning, but in particular, it contains domain knowledge in the field of structural health monitoring, so it can support engineers in the damage detection of concrete elements in a pre-failure state.

The approach presented by the authors for the dataset creation presented here moves the focus of the work to domain-specific aspects of the bridge industry. This dataset is intended to provide a basis for research into issues of computer vision and machine learning to fulfill the requirements of the bridge sector. It is the intention of the authors to provide the data science industry with such datasets that will allow for the creation of algorithms that will support the day-to-day work of the bridge engineer in accordance with aspects of structural work and standard requirements. In this approach, the authors consider a binary problem: there is a visible crack on the concrete surface representing the pre-failure state that can be observed by the human eye, or there is no such artifact on the concrete surface. Therefore, it is a binary problem, not one with an ad hoc assigned probability density distribution. However, the authors assume that a predicted crack course involving the result of a trained neural network based on the considered dataset, structural scheme and occurring loads may in the future lead to the indication of probable locations of cracks in concrete structures that are not visible on the surface. Such a situation, however, is not the subject of research at this stage.

The possibility of using this dataset to solve machine learning problems was verified by the Authors in the prototype solution presented in subsection “*Validation the possibility of using dataset for machine learning-based solutions”*.

The main goal of this dataset is the possibility of developing efficient and robust deep machine learning algorithms for both concrete structures crack classification and segmentation problems. This dataset can be used to train new models, as well as to verify the ability of already developed neural network models for early crack detection. Using the dataset to develop deep learning algorithms for early crack detection can be considered in many directions.

The dataset can be expanded both in terms of adding more images to increase the number of images, as well as in the direction of solving multi-class problems, for example, by adding a “bughole” or “formwork marks” class. Due to the size of the sub-image in the dataset, it can also be used to expand the dataset for crack inspection of also small but important elements of bridge structures such as deviator cracks. The next step in the dataset’s improvement will be a situation where the ML-based solution will not only support the bridge engineer’s work so that cracks observable to the human eye are not overlooked but will also allow automatic diagnosis of cracks that are identified only by machine means. The criterion for the dilation of an element can be based, for example, on the practice resulting from cartographic representations on maps (the unaided human eye is able to recognize 2 lines parallel to each other as separate if they are at least 0.2mm apart).

In connection with the development of BIM and Digital Twin technologies, the Authors are pointing out an important direction for the use of results obtained from training models based on the dataset presented. Addressing the problem of the crack occurrence for the pre-failure state is to link the identification of the crack, diagnose it, assign the occurrence of the damage in terms of geometric location on the structural model, and link the occurrence of the crack to the potential cause, i.e. the stress model resulting from the FEM analysis of the structural element. The model linking the type and attributes of a given structural element and the occurrence of a crack can be, for example, a BIM model in the IFC schema, in which for each structural element the attributes concerning the type of element, concrete class, exposure class, load capacity of the element can be included. It is also possible to link detected defects to data from SHM systems feeding the Digital Twin of the structure. This makes it possible to build models not only based on vision aspects, but also containing detailed information about the damaged element and their source.

Thus, this leads to significant development of AI-based services for SHM for pre-failure condition.

## Methods

This dataset was prepared on the basis of images taken in 2019–2022 by an experienced bridge engineer at construction sites and during inspections of engineering structures (bridges, viaducts, tunnels) in southern Poland.

This dataset contains images of cracks in elements made of reinforced concrete such as abutments, tunnel walls, concrete barriers, pillars. The damage images are differentiated by the cause of the cracking (e.g., excessive stresses, thermal and shrinkage stresses in young concrete). The origin of the images also differentiates the stage of the structure’s life cycle, because they were acquired from the stage of construction (at the stage when elements are only loaded by self-weight) to the stage when the structure is in use (at the stage when most design loads are applied). In addition, the structures were constructed by different contractors, which may indicate the different quality of work execution. Thus, this significantly expands the applicability of the dataset and its robustness to different boundary conditions.

In terms of exposure of photographed elements, this dataset covers:

- the full range of the concrete classes which are practically used for elements transferring loads characteristic for engineering structures,
- the full range of exposure to environmental conditions - images were taken for engineering structures elements transferring loads over inland watercourses, roads, as well as in tunnels,
- while the photos presented do not show damage for engineering structures in the marine area exposed to brine, this dataset is fully representative due to the fact that the photographed engineering structures elements are maintained in the winter season with brine, and the dataset also includes photos of tunnel structures exposed to groundwater,
- the dataset does not show damage to elements of the underwater engineering structures,
- the photos were taken in all four seasons and in the full range of pressure and humidity conditions characteristic for Central Europe.

The images were acquired using fixed-focus cameras without prior conditioning. It should be noticed that inspecting bridge structures often is connected with difficult access to structural elements (e.g., spans over rivers, high pylons) and the use of boom-type equipment in many cases. Documenting damages is in most cases required to create a condition assessment report. This makes that if the requirements for image acquisition conditions are set too high, it can significantly limit or even eliminate the possibility of image acquisition. For this reason, the Authors’ intention was to use equipment that is available for each engineer and can be used under any inspection conditions. It was also important for the Authors to work later with images that were acquired without the use of specialized and calibrated equipment and which may be of lower quality.

**Fig. 2:**
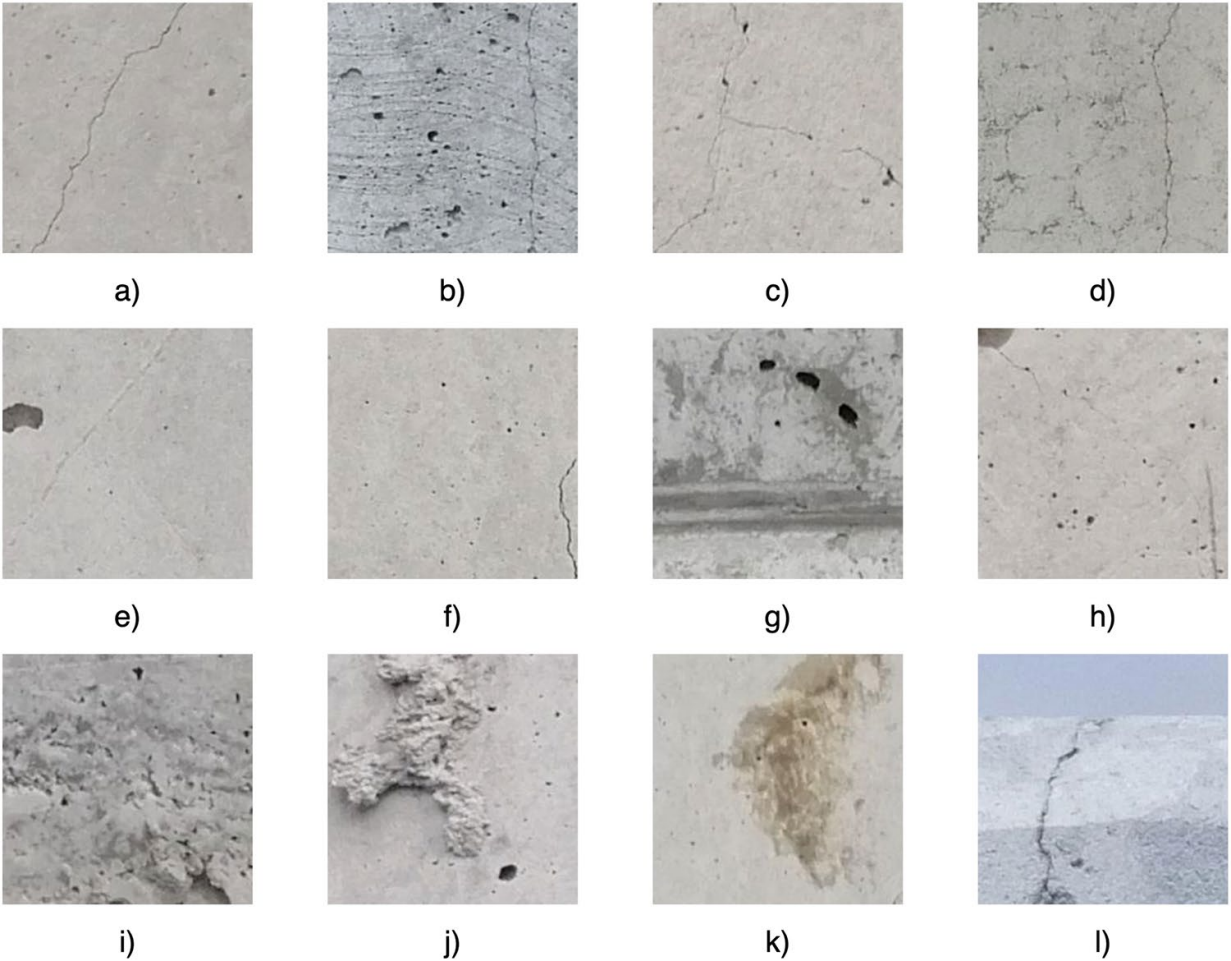
Examples of characteristic damage or complex concrete surface finish present in the dataset.

It should be noticed that a characteristic feature of this dataset is the very complicated nature of the concrete surface finish, including, for example, the presence of formwork marks (Fig. 2g), concrete troweling marks (Fig. 2b), dirt (Fig. 2h,k), and mechanical damage of the concrete surface (Fig. 2e). As the Authors’ experience has shown^19^, these damages can potentially be recognized as cracks and provide additional difficulties for damage detection by machine learning algorithms. However, they are very common and they occur independently of the work contractor (that is, their occurrence is unavoidable) therefore, from a practical point of view, they must be taken into account in the final solution. In the dataset presented here, the cracks can be located in different areas on the image, i.e. they do not necessarily occur in the central part of the image (Fig. 2f). In addition, the dataset also includes different colors of the concrete surface (e.g., Fig. 2b,c,d,k,l), bugholes (Fig. 2b,e,g,j) and background obstructions (Fig. 2l).

To create the dataset, images in .jpg format were used, in the resolution in which they were taken (i.e. 3464 × 4618 px to 3840 × 5120 px), without affecting the quality of the image.

**Fig. 3:**
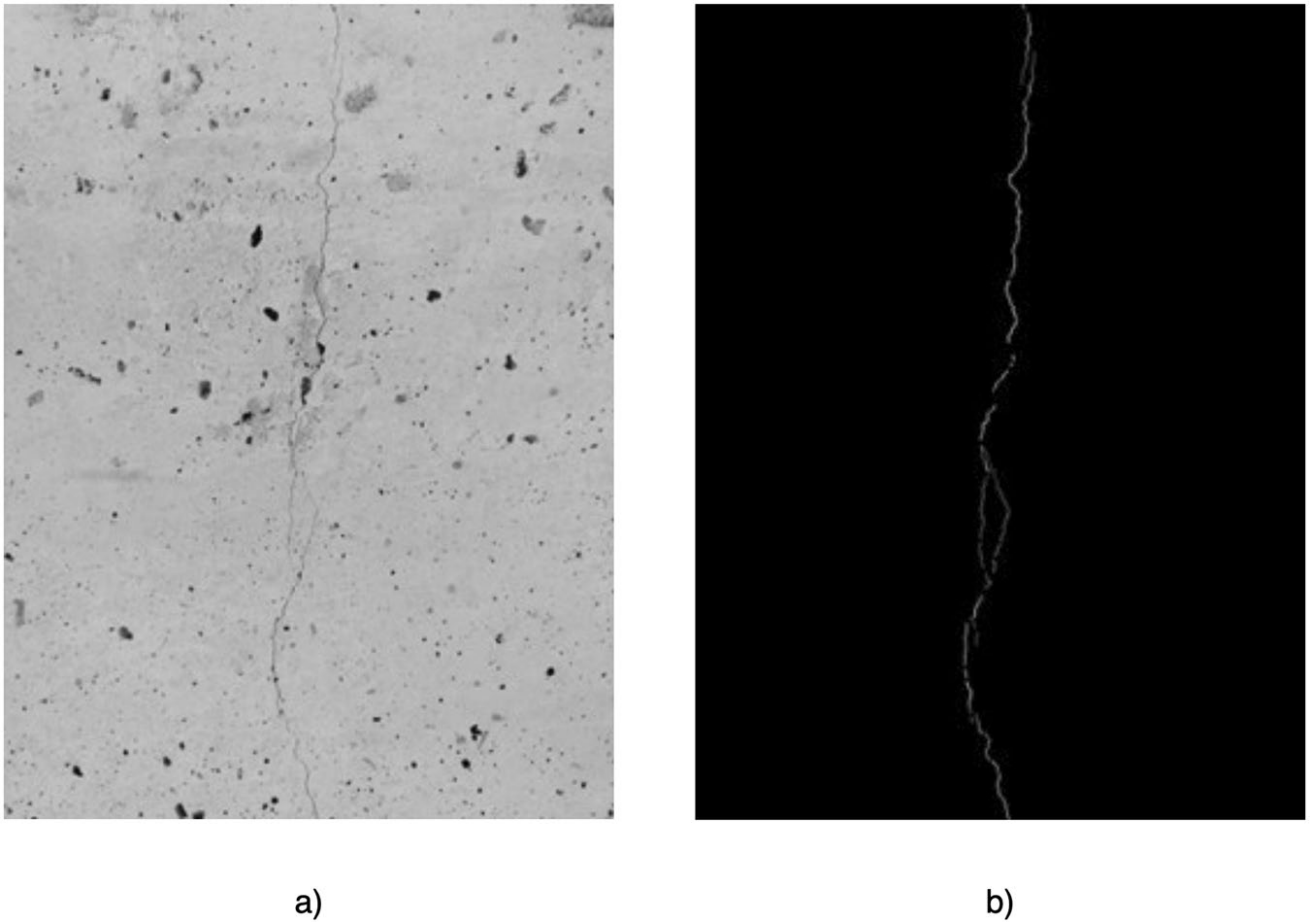
Example of full-resolution images before sub-image division (**a**) original image (**b**) segmentation mask.

**Fig. 4:**
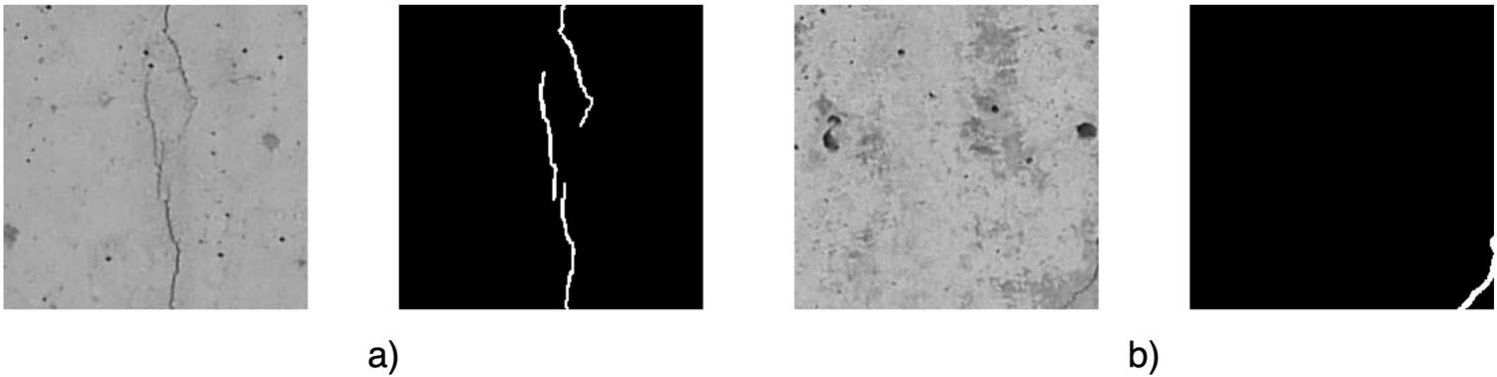
Examples of images and their segmentation masks after sub-image division (**a**) Crack in the center of the image (**b**) Crack in the corner of the image.

A mask was prepared for each image in GIMP by manually selecting the pixels representing the damage by an experienced engineer. A detailed description of the annotation process is presented in the subsection “*Validation of correct class assignments to pixels”*. Each mask was exported to a .png file. An example of the image and its corresponding mask is shown in Fig. 3. The image-mask pairs were then divided into sub-images according to the defined sub-image size (i.e., 224 × 224 px), without overlapping sub-images. In order to eliminate errors related to the export of the mask from GIMP (i.e., with the possibility of having in the mask, in addition to pixel values of 0 and 255, also values close to 0 and 255, such as 1, 4, 253), a thresholding operation was performed for each mask sub-image to receive a binary image. The sub-images of images and masks prepared in this way were used to create a segmentation dataset. An example image and its corresponding mask after dividing into sub-images is shown in Fig. 4.

The classification dataset was created based on the segmentation dataset. The adopted approach was that the image for which there are pixels indicating crack in the mask is automatically classified as “Cracked” and “Uncracked” for images in which there are no crack pixels in the mask. It is important to note that after the process of automatic classification of the image assignment to each class, the correctness of the algorithm was manually verified in the context of the proper image assignment to each class. For details of this classification validation, see the subsection *“Validation at the stage of creating the classification dataset”*.

**Fig. 5:**
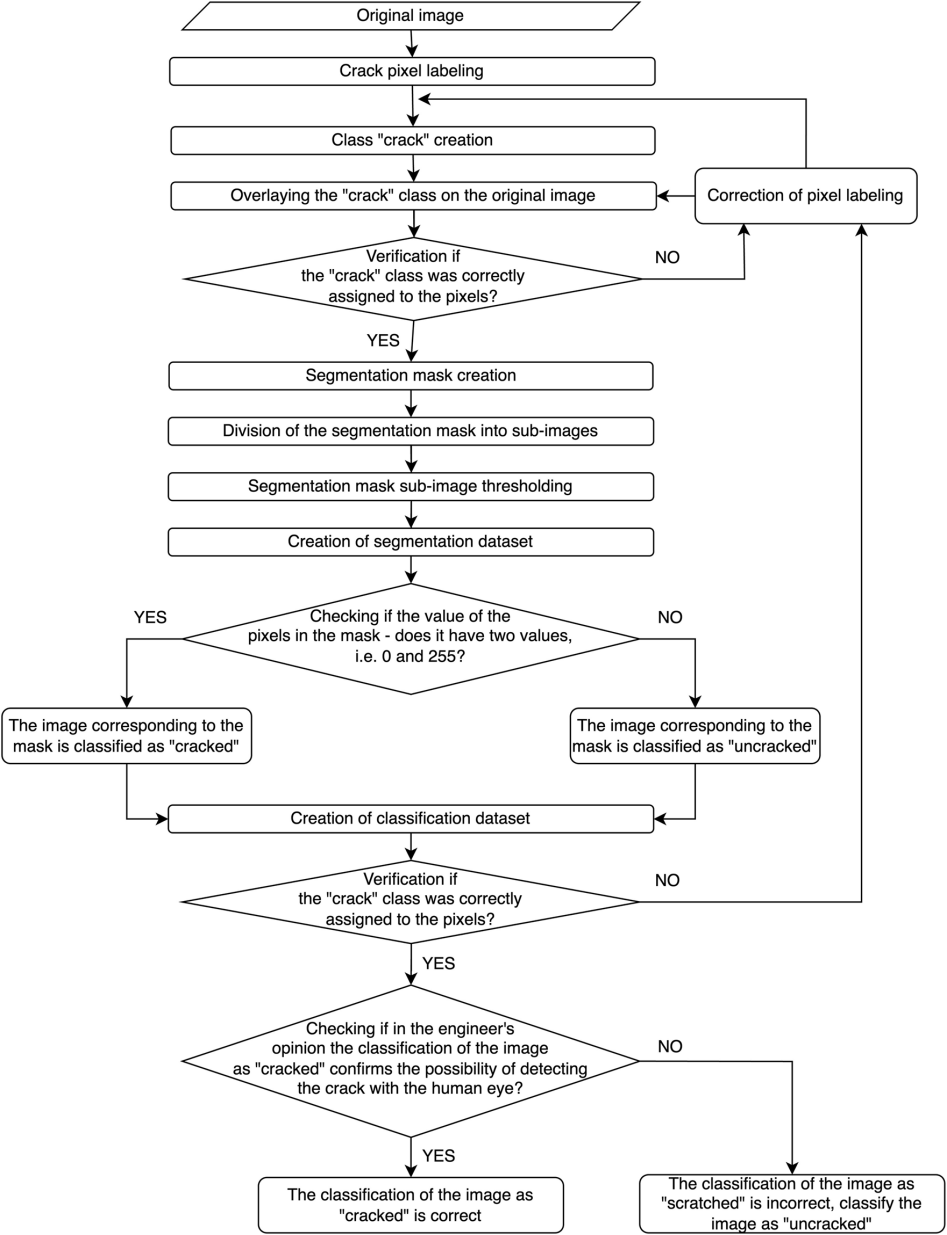
Schema of creating segmentation and classification dataset.

A schematic representation of the dataset creation process is shown in Fig. 5.

## Data Records

The dataset titled “*NCCD-PF - A pre-failure narrow concrete cracks dataset for engineering structures damage classification and semantic segmentation*” is publicly available in the Zenodo data repository^22^. The dataset is divided into two main folders, namely “Dataset_for_classification” and “Dataset_for_semantic_segmentation”.

The classification dataset contains two subfolders, namely “Cracked” and “Uncracked.” Each sub-folder contains 224 × 224 px images, saved in .jpg format. The images have been reshuffled and numbered starting from 0. The file naming convention is as follows: for images in “Cracked” subfolder, the filename is “image_C_X.jpg” and the filename of the image is “Uncracked” subfolder is “image_UC_X.jpg “, where X is the next image number in the subfolder. The number of images in each class is shown in Table 1.

**Table 1:** Parameters and number of data in the dataset.

		Dataset for classification	Dataset for semantic segmentation
Data format		.jpg (images)	.jpg (images), .png (masks)
Images size		224 × 224 px (images)	224 × 224 px (images)
			224 × 224 px (masks)
Number of images	Cracked	668	—
	Uncracked	4720	—
	Total	5388	5388

The dataset for semantic segmentation contains two subfolders, namely “Images” and “Masks.” The “Images” folder contains images in.jpg format, and the “Masks” folder contains binary masks in .png format (where white color indicates damaged pixels and black color indicates background pixels). As with the classification dataset, the images (and corresponding masks) have been reshuffled and numbered starting from 0. The file naming convention is as follows: for images, the filename is “image_X.jpg” and the filename of the corresponding mask is “image_X_mask.png”, where X is the next image number in the dataset. The number of images in each class is shown in Table 1.

It should be noted that each dataset has its own universal numbering of images (i.e., image #1 in the classification dataset does not necessarily correspond to image #1 in the semantic segmentation dataset).

## Technical Validation

Due to the complexity of the problem with the detection of concrete element cracks (Section *“Background & Summary”* and *“Methods”*), the development of datasets for crack detection of concrete elements is not an obvious and straightforward issue. It requires domain knowledge of the bridge structures’ work and their diagnostics. The correctness of the approach to dataset creation was verified in two stages, i.e., making assumptions about the conditions of image acquisition and at the stage of image processing, where three parts were distinguished, i.e., the creation of a segmentation and classification dataset as well as the correctness of class assignments to pixels. In addition, the possibility of using the dataset to build a solution based on machine learning algorithms was verified.

### Validation at the stage of image acquisition

The images that were the basis for the dataset were taken by an experienced bridge engineer. Such cracks of individual concrete elements were selected which are most characteristic for these elements in terms of location and cause of appearance. The moment of crack appearance was also taken into account, i.e. the dataset contains both cracks appearing already at the construction stage (whose condition may get worse under further loads), as well as those appearing at the structure’s use stage (under most design loads).

The selection of representative damages considered, in particular, damages such as:

- Cracks caused from non-uniform ground settlement – characteristic for foundations, abutments, pillars, retaining walls, tunnel walls;
- Cracks caused by casting in stages – where the shrinkage of the fresh concrete is restrained by the hardened concrete cast in the previous stage. E.g. vertical cracks in the webs of box girders;
- Cracks in prestressed structures, including cracks caused by corrosion of tendon anchorages, corrosion of tendons, decompression effects or not enough reinforcement in the anchorage zone – characteristic for girders;
- Cracks caused by corrosion of steel reinforcement – as the rust builds up, tensile stresses increase, causing cracking of concrete. Dangerous in particular because of the risk of concrete spalling and exposure of corroded reinforcing bars – characteristic for abutments, piers, girders;
- Cracks caused due to increased shear stress – appearing near supports like wall or pillar;
- Cracks due to increased bending stress near the center of the element’s span (in extremal bending moment zones) – characteristic for girders;
- Cracks due to compression failure – appearing at the top of the element when it is over-reinforced;
- Cracks caused by plastic shrinkage, i.e., too fast evaporation of moisture from the setting concrete – characteristic for slabs, footpaths.

In order to show the complexity of each problem, the following paragraphs discusses in more detail one of the possible causes of cracks, i.e., damage in the lower part of the front wall of the bridge abutment.

In the case of bridge structures, we are dealing with a special type of cracking, which is characteristic for massive structures and occurs already at the construction stage – when the strength of concrete is much lower than designed and when there are no service loads yet. Examples of massive elements in bridges are foundation slabs, pillars, pylon, or – as in the case of the dataset presented here – abutments.

We can determine the massiveness of an element by comparing the ratio of the element’s surface area to its volume. This is related to the cement hydration processes that occur in concrete elements. So in the case of massive elements, in the process of cement hydration during the setting of concrete, the ratio of surface area (through which the heat of hydration is dissipated) to volume (in which heat is released) is small, which is one of the main problems in the technology of massive structures.

In the case of abutment walls, we are dealing with the constraint on the freedom of deformation by external links. Abutment walls are made after the foundation has hardened and cooled, hence the maturation of the wall occurs when the bottom of the wall is restrained in the foundation and there is no possibility of deformation. Thermal-shrinkage cracks of abutments are vertical cracks that begin above the wall-foundation joint and disappear at the top of the wall. They are a danger, because under further loads (by structural elements or traffic) cracks can propagate through the entire thickness of the concrete. As a consequence, the monolithicity of the structure may be lost and its static scheme may be changed. In addition, the crack is that place through which water can penetrate into the element. This, in turn, can promote the development of corrosion processes in steel and concrete.

As this extended description of a single crack for a single element and a single cause shows, the problem of crack detection is a very multi-threaded problem.

Therefore, this article often emphasizes the importance of not only having information about the occurrence of the crack, but also the importance of being able to assess the threat it poses to the damaged element and, consequently, to the entire structure (in relation to the probable cause and location of the damage).

### Validation at the stage of creating the semantic segmentation dataset

Analyses conducted by the Authors have shown that in the case of narrow cracks and complex finish of the concrete surface, which is the background for the crack, the use of automatic methods to support crack detection (e.g. edge detection filters, sharpening operations, thresholding operations) are not effective.

This is confirmed by examples of the use of the above-mentioned filters or operations that affect the quality of the image, which are popular for crack detection problems^23,24^. It should be noticed that in the cases identified in the literature, we are mainly dealing with wide crack widths and a uniform concrete surface or wide crack widths and a complex concrete surface^20^, so that these methods can provide high detection results.

**Fig. 6:**
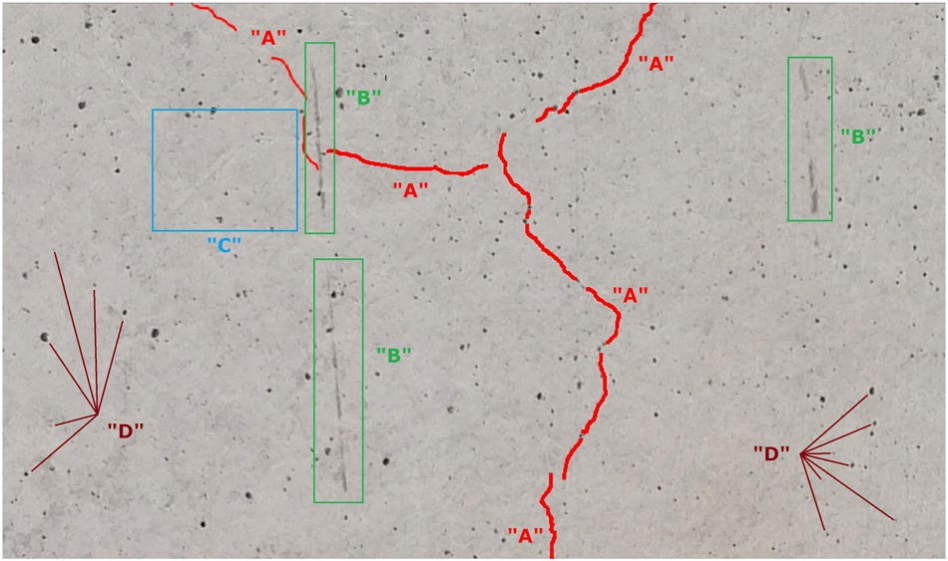
Example of concrete surface appearance. “A” – crack, “B”. – dirt, “C” – mechanical damage, “D” – bugholes (in order to ensure the image’s clarity, only some are marked).

Figure 6 shows an example of a concrete surface finish with a narrow crack (marked on the image as “A”) as well as non-crack elements (dirt – marked as “B”, mechanical damage - marked as “C”, bugholes – marked as “D”). Table 2 shows examples of the application of various automatic methods for crack detection and mask creation. The filters were applied to the same image as shown in Fig. 6. The table shows the result of applying the filter to the image (column “Image of the crack”) and the applied correct crack (column “Image of the crack with the mask applied”). For narrow cracks and complex concrete surface finishes, the use of these methods makes it possible to identify the main direction of the crack (ex. Sharpening operation), but is ineffective for smaller widths (ex. Sobel filter, Gauss filter, thresholding operation). As important, these operations do not provide the domain knowledge to determine whether a particular crack could pose a potential threat to the structure in the future and should be included in the monitoring. In addition, the use of these operations makes visible, for example, mechanical damage or dirty concrete with colors similar to cracks, which do not pose any threat to the structure and should not be considered as crack. However, this requires domain knowledge.

**Table 2:** Example of filter and operations applied on crack images from dataset.

Type of filter or operation used	Image of the crack	Image of the crack with the mask applied
Original image		
Sharpening operation		
Sobel filter		
Gauss filter		
Thresholding (170–255 px)		
Thresholding (185–255 px)		

Therefore, it is not possible to use automatic methods to support narrow crack detection (e.g. edge detection filters, sharpening operations, thresholding operations) to create masks for narrow cracks occurring on a concrete surface with a complex finish. Thus, automatic methods do not allow to detect crack and to develop reliable datasets. For the dataset presented in this paper, after the images were taken, each pixel was manually annotated by an experienced bridge engineer in GIMP and received an assignment to a crack or background class. This made it possible, on the one hand, to isolate the non-crack damage and, on the other hand, to identify those crack pixels that should be further analyzed at the stage of applying machine learning algorithms.

### Validation at the stage of creating the classification dataset

As explained in the *“Methods”* section, the creation of the classification dataset used an automatic algorithm based on the created segmentation masks. Images whose mask contained a crack class were automatically classified as “Cracked,” and images that did not fulfill this requirement (i.e. having only background class pixels) were classified as “Uncracked.” Classified images were placed in the corresponding class folders in the classification dataset.

**Fig. 7:**
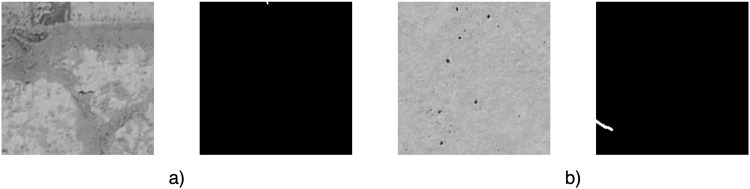
Examples of manual verification of correct class assignment (**a**) final section of crack (**b**) crack with very small width.
- The crack is larger than in the case of a) (Fig. 7b) – in terms of the pixel percentage of the crack compared to the background on the segmentation mask – but has such a small width, that it is difficult to see with the human eye and the classification as a crack without having the context of the whole crack’s progression. In the engineer’s opinion, having the role of classifying such an image (without having the support of a segmentation mask), it would not be possible to recognize a crack on it. For the purpose of image classification, it was decided to change the classification of such an image to “Uncracked”.

After the automatic classification process of the images was completed, the correctness of their assignment to each class was verified manually. This was also supported by the analysis of the created segmentation masks. In particular, attention was paid to two cases observed in the images:

- There is a final section of crack on the image (Fig. 7a) – on the segmentation mask, the percentage of crack pixels is small compared to the background pixels. In the engineer’s opinion, such a small section of crack would not be detectable in the image. In this case, the decision was made to change the classification of this kind of image to “Uncracked”.

### Validation of correct class assignments to pixels

The mask preparation was based on the domain knowledge of the engineers who carried out the inspections of the structures. The mask preparation was done manually by selecting pixels. In particular for the mask creation, other machine learning and deep machine learning methods were not used.

The correctness of class assignments to pixels was made by providing consistent annotation in terms of content and by verifying possible labeling of accidental inclusion of non-crack pixels or missing crack pixels.

The criterion for assigning a group of pixels to a crack class was:

1. Identifying the pixel as representing a discontinuity in the concrete surface.
2. Eliminating this pixel based on engineering knowledge and site context as a pixel representing dirt, formwork marks, etc. - that is, other changes in the concrete surface structure that do not represent potential structural damage.

Regarding providing consistency of annotation in terms of content before annotating the crack pixels, the bridge engineer received two documents: a rule of what should be annotated and a set of examples that contained images corresponding to the elements of the confusion matrix, i.e. annotations understood as False Positive, False Negative, True Positive, True Negative. At the stage of verifying the pixel annotation correctness, another engineer who did not perform the annotation process received the annotation results and annotation rules. Based on the rules, that engineer performed a quality check.

Regarding the possible labeling of accidental inclusion of pixels without cracks or missing crack pixels the correctness of the pixel label assignment was verified at two stages - in the first step at the stage of creating the segmentation mask and in the second step at the stage of creating the classification dataset. Two aspects were verified:

1. Whether all crack pixels were classified correctly. The authors verified in the first step by overlaying the segmentation mask (strictly speaking, the “crack” class) on the original image and verifying that all pixels in the crack course were covered by the “crack” class. In the second step, each sub-image of the mask was analyzed for pixels not marked as a crack in the crack course (discontinuity within the crack).
2. Whether random pixels marked as cracked were found. In the first step, the verification was done by checking the mask image to check if there are any pixels labeled with the class “background” in the range of pixels labeled with the class “crack” outside the crack course. In a second step, each mask sub-image was analyzed for the visibility of incorrectly labeled crack pixels outside its course. If any errors were identified in any aspect and in any step, the pixel labeling was corrected, and the segmentation and classification dataset was created again, based on the corrected mask.

### Validation the possibility of using dataset for machine learning-based solutions

**Fig. 8:**
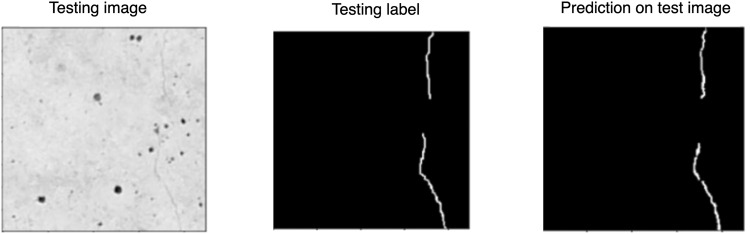
Results of the Authors’ dataset-based prototype solution.

**Fig. 9:**
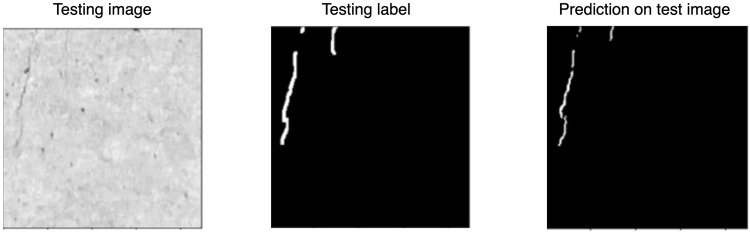
Results of the Authors’ dataset-based prototype solution.

In order to verify the standard of dataset delivery (including image size, dataset size) and to verify that this dataset is valuable for machine learning, the authors built a prototype solution. A U-Net model was trained from scratch on a subset of 791 images of size 224 × 224 px. The solution showed satisfactory results for pixel classification in detecting narrow cracks, as shown in Figs. 8, 9.

## Data Availability

No custom code was generated for this work.
